# Supplementary material for: The Association of Minimally Invasive Surgical Approaches and Mortality in Patients with Malignant Pleuropericarditis—A 10 Year Retrospective Observational Study
Source: Medicina (Kaunas). 2022 May 27;58(6):718. doi: 10.3390/medicina58060718 (PMC9229806; doi:10.3390/medicina58060718)
Supplement: Supplementary file 1 [file medicina-58-00718-s001.zip › medicina-1672585-supplementary.pdf]

Supplementary materials

Table S1. Distribution of all variables in the sample

Variables	All sample (N = 338)
Patients' characteristics and diagnosis types	
Event	
Alive/Censored	28 (8.28%)
Death	310 (91.72%)
**Sex**	
Males	164 (48.52%)
Females	174 (51.48%)
Age (y)	60.92 (13.58), 19-94
Imaging diagnosis	
Echocardiography	39 (11.54%)
Computed tomography	299 (88.46%)
Clinical Diagnosis	
Pericarditis and right pleural effusion	141 (41.72%)
Pericarditis and left pleural effusion	162 (47.93%)
Pericarditis and bilateral pleural effusion	35 (10.36%)
Surgery type	
Thoracoscopic pleuropericardial window	288 (85.21%)
Subxiphoid pleuropericardial window through mediastinoscopy	50 (14.79%)
Comorbidities	
No comorbidity	71 (21.13%)
Hypertension	137 (40.77%)
Diabetes	26 (7.74%)
Atrial fibrillation	15 (4.46%)
Renal insufficiency	17 (5.06%)
Heart failure	54 (16.07%)
Hepatic insufficiency	8 (2.38%)
Chronic obstructive bronchopneumpathy	8 (2.38%)
Presence of pericardial tamponade	
Yes	35 (11.83%)
No	298 (88.17%)
Presence of hypodiastolia	
Yes	
No	
The patients' cancer types	
Malignancies	
Lung Cancer	181 (53.55%)
Esophageal/Gastric Cancer	6 (1.78%)
Leukemia/Lymphoma	43 (12.72%)
Brest Cancer	55 (16.27%)
Pleural Mesothelioma	21 (6.21%)
Ovarian Cancer	11 (3.25%)
Cervical Cancer	11 (3.25%)
Other types of cancers (Sarcoma, Renal, and Colorectal Cancer)	10 (2.96%)

Variables	All sample (N = 338)
Metastases	
No other metastases	308 (91.12%)
Heart	2 (0.59%)
Liver	6 (1.78%)
Peritoneum	5 (1.48%)
Bone	13 (3.85%)
Upper kidney	3 (0.89%)
Kidney	1 (0.30%)
Pleurodesis treatment and recurrence rate	
Pleurodesis treatment	
Talc	49 (14.50%)
Betadine	289 (85.50%)
Recurrence of pleuropericarditis	
Recurrence	10 (2.96%)
No recurrence	328 (94.04%)

Abbreviations: N, number; SD, standard deviation; y. years; N = 338.
